# Integration of deep learning and habitat radiomics for predicting the response to immunotherapy in NSCLC patients

**DOI:** 10.1007/s00262-024-03724-3

**Published:** 2024-06-04

**Authors:** Weimin Caii, Xiao Wu, Kun Guo, Yongxian Chen, Yubo Shi, Junkai Chen

**Affiliations:** 1 Department of Emergency, Wenzhou Hospital of Integrated Traditional Chinese and Western Medicine, Wenzhou, 325000 China; 2 Department of Gastroenterology and Hepatology, The First Affiliated Hospital of Wenzhou Medical University, Wenzhou, 325000 China; 3 Department of Chest Cancer, Xiamen Second People’s Hospital, Xiamen, 36100 China; 4 Department of Pulmonary, Yueqing People’s Hospital, Wenzhou, 325000 China

**Keywords:** Immunotherapy, Lung cancer, Clinical durable benefit, Deep learning, Habitat radiomics

## Abstract

**Background:**

The non-invasive biomarkers for predicting immunotherapy response are urgently needed to prevent both premature cessation of treatment and ineffective extension. This study aimed to construct a non-invasive model for predicting immunotherapy response, based on the integration of deep learning and habitat radiomics in patients with advanced non-small cell lung cancer (NSCLC).

**Methods:**

Independent patient cohorts from three medical centers were enrolled for training (*n* = 164) and test (*n* = 82). Habitat imaging radiomics features were derived from sub-regions clustered from individual’s tumor by K-means method. The deep learning features were extracted based on 3D ResNet algorithm. Pearson correlation coefficient, *T* test and least absolute shrinkage and selection operator regression were used to select features. Support vector machine was applied to implement deep learning and habitat radiomics, respectively. Then, a combination model was developed integrating both sources of data.

**Results:**

The combination model obtained a strong well-performance, achieving area under receiver operating characteristics curve of 0.865 (95% CI 0.772–0.931). The model significantly discerned high and low-risk patients, and exhibited a significant benefit in the clinical use.

**Conclusion:**

The integration of deep-leaning and habitat radiomics contributed to predicting response to immunotherapy in patients with NSCLC. The developed integration model may be used as potential tool for individual immunotherapy management.

## Introduction

Non-small cell lung cancer (NSCLC) is one of the main cause of cancer-related mortality in global [1]. Recently, Immunotherapy, the immune checkpoint inhibitors such as PD-1 or PD-L1, has revolutionized the treatment paradigm for malignant cancer, establishing itself as the novel benchmark for managing locally advanced and metastatic non-small cell lung cancer (NSCLC) patients [2]. Numerous studies have demonstrated that the efficacy of immunotherapy on improving long-term survival rates when used as a monotherapy or in integration with other therapies [3–6]. Despite these advancements, only a part of patients, ranging from 20 to 50%, exhibits responsiveness to the therapy [7, 8]. The uncommon patterns of immunotherapy response, such as delayed response or pseudo-progression, makes traditional response prediction approaches obsolete. Additionally, there is a potential risk of immune-related adverse events which influences the patients' prognosis [9].

The identification of biomarkers with immunotherapy response power has become increasingly important for effective condition monitoring. Various biomarkers, including PD-L1 expression and tumor mutational burden, have raised researcher’s attention. And their relationship to immunotherapy response have been reported in previous studies, though conclusion differs [10, 11]. Moreover, the reliability of these biomarkers may be influenced by tumor heterogeneity, as they mostly depend on biopsy samples that do not reveal the full spectrum of the tumor microenvironment.

Due to the significant heterogeneity of malignancies, tumors present diverse microenvironments and microstructures [12–15]. Radiomics, an approach that extracts high throughput features to classify disease condition using machine learning techniques, holds promise for personalized medicine decision-making. However, conventional radiomic analysis generally focuses on the entire tumor, ignoring the sub-regional phenotypic variations within the tumor [16]. Recently, a novel method known as 'habitat' has emerged, which partitions tumors into sub-regions by identifying grayscale voxels with similar imaging characteristics [16]. This approach has demonstrated potential in enhancing the differentiation of tumor heterogeneity [17, 18]. Furthermore, recent developments in deep learning have revealed that neural networks can automatically extract radiomic features without the need for human intervention, leading to improvement of predictive performance [19, 20].

Additionally, the integration of multimodal information, such as deep learning features and habitat radiomic features, could offer tumor-specific multi dimension data for better immunotherapy management [21, 22].

The present study was conducted to explore the potential improvement in predictive accuracy through the integration of imaging data during the early stages of treatment. The support vector machine (SVM), was developed and subsequently validated on an external dataset to predict the possible benefit of immunotherapy in NSCLC patients at 6 months following treatment commencement.

## Method

### Patient selection

**Fig. 1:**
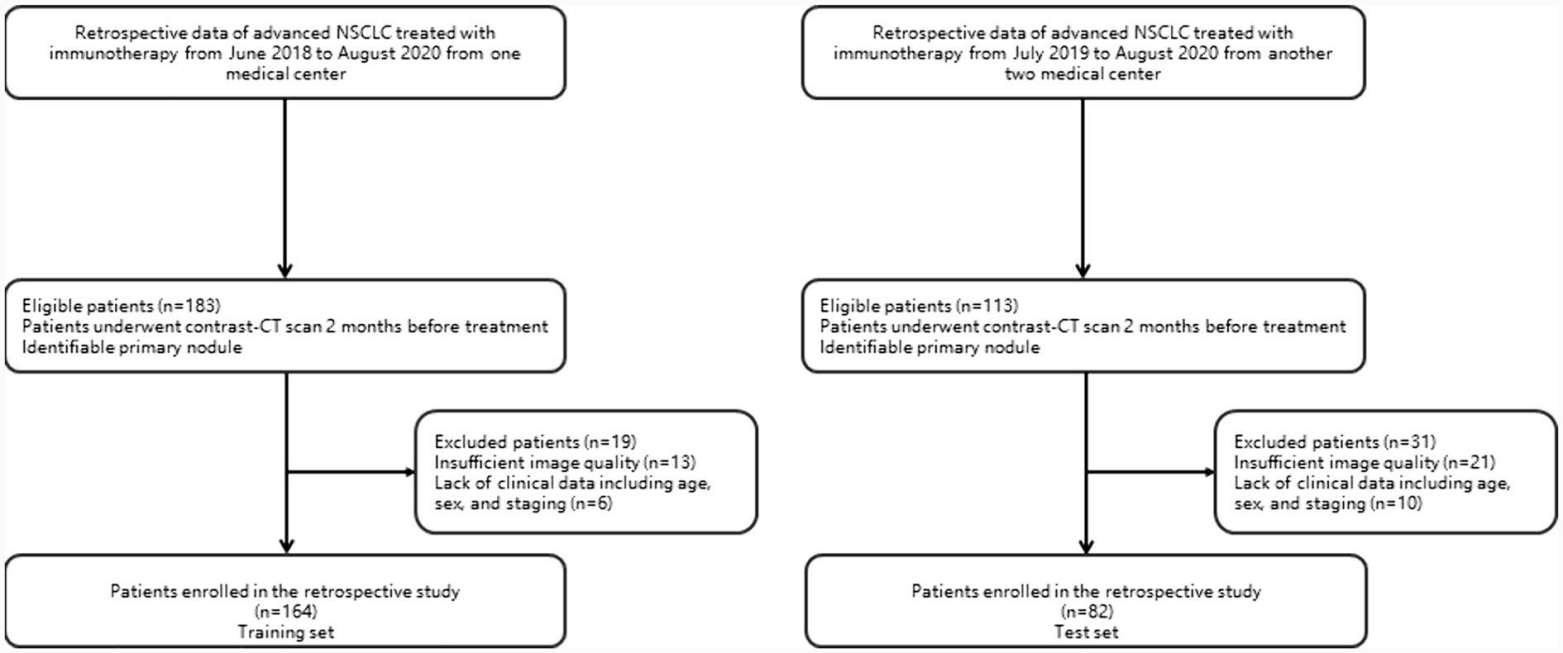
Flowchart showing the inclusion and exclusion criteria considering the endpoint PFS6. Details of the number of patients in the training and independent test set are provided

A total of 246 patients diagnosed with NSCLC treated with immune checkpoint inhibitor between January, 2018, and May, 2020 were enrolled at the Wenzhou Medical University First Affiliated hospital, Yueqing people’s hospital, Xiamen Second hospital in this study, as depicted Fig. 1. This study has been approved by their institutional reviews board. Written informed consent was waived because the data used in this manuscript were anonymized by removing personal information and its nature of retrospective design. All enrolled patients met the following inclusion criteria: (1) pathological diagnosis of NSCLC; (2) chest contrast CT examinations were available. Patients were excluded if: (1) Basic clinical data (such as age, sex) were missing; (2) preoperative treatment or the time between CT examination and subsequent surgery exceeded 1 month; (3) CT images were of low quality; and (4) molecular testing results were difficult to determine.

Finally, the patients from Wenzhou Medical University First Affiliated hospital were used as training set (*n* = 164), the remaining patients from two other institutions were utilized as test set (*n* = 82).

### Clinical endpoint

The primary objective of this study was the durable clinical benefit, as defined by progression-free survival (PFS). This metric represents the interval from the start of the first immunotherapy cycle until the occurrence of death, disease progression, or the last follow-up. Disease progression was judged by the individual’s overall clinical condition and the response evaluation criteria in solid tumors [23, 24], derived from imaging evaluations. Patients who exhibited a durable clinical benefit with a PFS exceeding 6 (PFS6) months were classified as responders [25], whereas the remaining were labeled as non-responders. Patients with censored data 6 months post-treatment were omitted from the analysis.

The secondary clinical outcome, overall survival (OS), was defined as the duration in months from the commencement of immunotherapy to the occurrence of death or the last follow-up date.

### Image acquisition and Tumor segmentation

All patients received a CT scan within a one-month period prior to the administration of immunotherapy. The contrast CT images were obtained after contrast injection during an inspiratory breath-hold, in accordance with the established protocol for contrast-enhanced CT of the chest.

Two experienced radiologists were tasked with delineating the border of tumors. The definitive VOIs were derived from their mutual consensus. In the event of a disagreement, a third radiologist was consulted to provide opinion. The delineation was processed using the ITK-SNAP (Version 4.0).

### VOI delineation and sub-region clustering

Habitat analysis employs entropy and voxel metrics extracted from CT imaging to categorize volumes of interest (VOIs) into different sub-regions [26–29]. The count of voxels for each VOI were derived by a conventional approach. Entropy values are obtained from CT images.

**Fig. 2:**
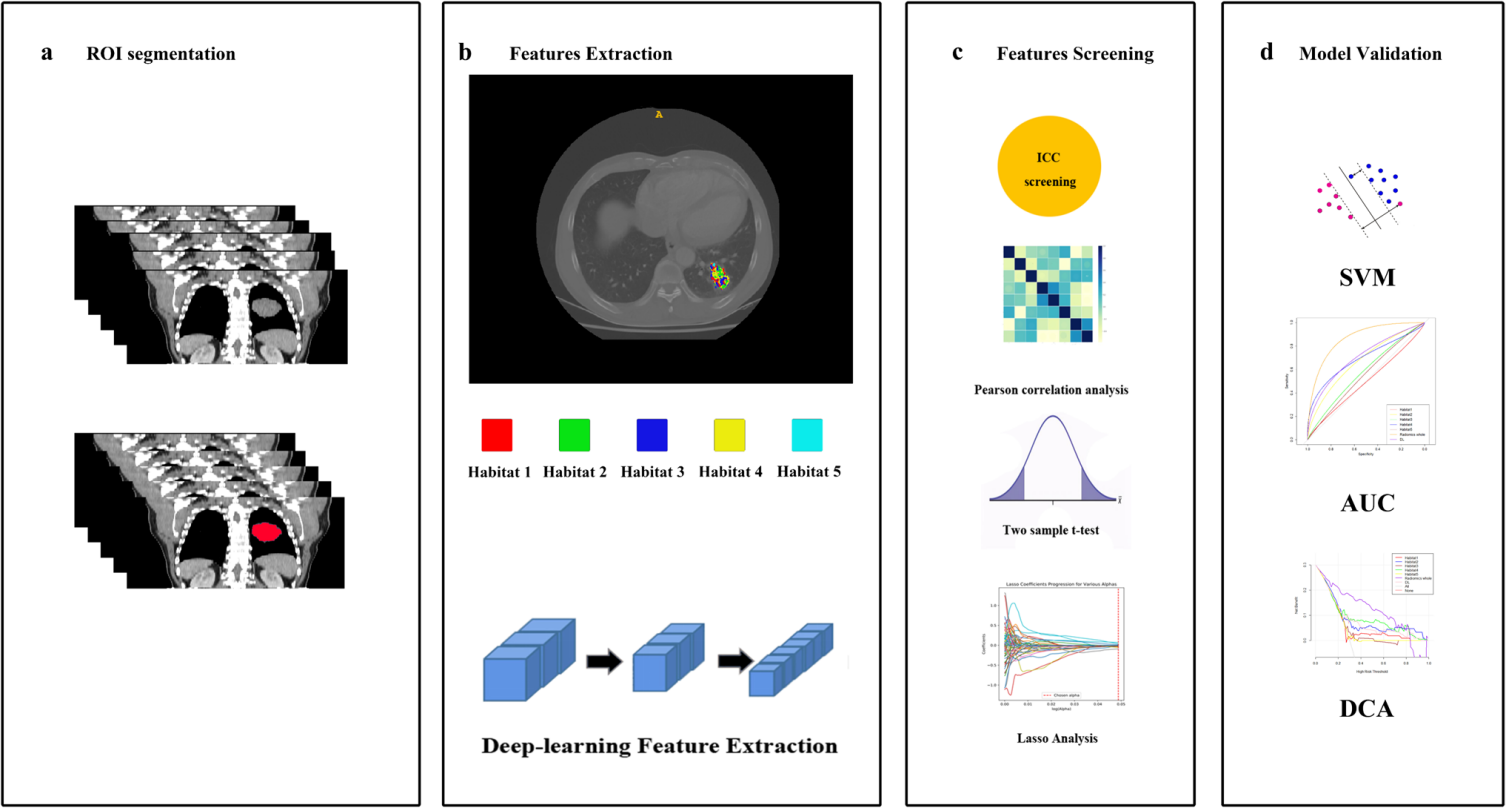
Implementation workflow of the ensemble models

The K-means clustering algorithm was utilized to label the VOI of each patient, leading to the formation of multiple habitats. Euclidean distance, based on voxel and entropy values, was used to identify the relationships among samples. We tested the number of habitats ranging from 2 to 10. To determine the optimal *k* value, we employed the Consensus Cluster Plus approach. The optimal *k*-value was crucial for determining the suitable number of clusters within the patients, as depicted in Fig. 2. Finally, our analysis determined the optimal *k*-value to be 5. Subsequently, we used the Python to extract each patient's radiomics information, and labeled each sub-regions named habitat 1, habitat 2, habitat 3, habitat4, habitat5.

### Feature selection and model development

To tackle differences in imaging features due to changes in the reconstruction layer thickness and pixel size, we resampled the images to a dimension of 1 × 1 × 1 m^3, and normalized them to a grayscale range of 0–255. The PyRadiomics [30] Python package was applied to independently extract radiomics features from six distinct habitats: habitat 1, habitat 2, habitat 3, habitat 4, habitat 5, and the entire tumor. This Python package is in compliance with the Imaging Biomarker Standardization Initiative [31].

On the other hand, we also used a 3D ResNet model as the CT image feature extractor to obtain deep learning features. The deep learning feature extraction module processed the region of interest (ROI) as input. When compared with traditional 2D image analysis, the 3D ResNet takes 3D context into consideration, thereby capturing more comprehensive image information from different level of one solid tumor, and make better decision. Finally, we obtained deep learning features from the whole tumor.

As for the features selection, at first, the features with an intraclass correlation coefficient (ICC) below 0.75 were filtered out. Subsequently, the remaining imaging features were underwent Z-score standardization to normalize the data to a mean of zero and a variance of one. Then we assessed the inter-feature relationships using the Pearson correlation coefficient. In instances where the correlation over the threshold of 0.9, we only kept one feature from each pair of highly correlated features to avoid redundancy. Finally, we applied two-sample *T* test and the least absolute shrinkage and selection operator (LASSO) regression model to further refine the remaining features in the training dataset.

A support vector machine (SVM) classification model was constructed utilizing features from each single habitat, the entire tumor, and deep learning features with five-fold cross-validation. Then, also fused each habitat tumor radiomics features with deep learning features, and fused all habitat tumor radiomics features with deep learning features as a combination to predict the risk of clinical endpoint.

### Model explanation

The SHAP (SHapley Additive exPlanations) algorithm, based on Cooperative Game Theory and local explanations, was utilized to elucidate the contribution of each feature to the final prediction output of the SVM model. By assigning an importance value to each feature for every prediction, SHAP visualized the contribution of each feature on the model's prediction, indicating how it either increased or decreased the likelihood of a particular outcome. SHAP analysis was performed using the KernelExplainer package (version 0.40.0) in Python (Version 3.7.0).

### Statistical analyses

Statistical analyses in this study were conducted using R software (version 4.0.2). Univariate analyses, comprising chi-square tests, t tests, and Mann–Whitney *U* tests were performed according to the normality of variables. Intraclass correlation coefficient (ICC), least absolute shrinkage and selection operator (LASSO), receiver operating characteristic (ROC) analysis, survival analysis, and decision curve analysis (DCA) were carried out using R statistical software. A two-sided p value of less than 0.05 was deemed to indicate statistical significance.

## Results

### Baseline clinical characteristic

A total of 246 patients from three institutions with NSCLC had received immune checkpoint inhibitor treatment and met the other inclusion criteria were finally included in our study. The mean age was 66.4 ± 8.4 [standard deviation]. Men were predominant (*n* = 215, 87.4%). There were 16 patients were never smoker. Regarding our study, we found the following: 59.3% included patients responded to immune checkpoint inhibitor after 6 months therapy. The most histology type was the adenocarcinoma. And 93.5% enrolled patients (*n* = 230) were current or former smokers. The therapy included monotreatment (*n* = 134, 54.5%), treatment with chemotherapy (*n* = 26, 10.6%), treatment with radiotherapy (*n* = 13, 5.3%) and combined immunological agents (*n* = 41, 16.7%), the other detailed demographic and clinical characteristic are shown in Table 1. Patients were split into training and test set based on their source. One hundred and sixty-four patients were used for model training, and 82 patients were utilized for external validation. There were no significant differences between the training and test set in demographic and clinical characteristics variables.

**Table 1:** Characteristics of NSCLC patients in training and external test cohorts

	All (*n* = 246)	Training set (*n* = 164)	Test set (*n* = 82)	*P* value
Age	66.4 ± 8.4	66.7 ± 8.4	65.7 ± 8.4	0.39
Sex: male	215 (87.4)	143 (87.2)	72 (87.8)	0.89
Height (cm)	165.1 ± 6.5	165.0 ± 6.6	165.2 ± 6.3	0.84
Weight (kg)	58.4 ± 10.4	57.6 ± 10.2	60.0 ± 10.7	0.09
BMI	21.4 ± 3.4	21.2 ± 3.5	21.9 ± 3.3	0.10
Smoking				0.12
Current smoker	38 (15.4)	27 (16.5)	11 (13.4)	
Former smoker	192 (78.0)	130 (79.3)	62 (75.6)	
Non-smoker	16 (6.5)	7 (4.2)	9 (11.0)	
Histopathology				0.60
LUSC	44 (17.9)	27 (16.5)	17 (20.7)	
LUAD	178 (72.4)	122 (74.4)	56 (68.3)	
Other	24 (9.7)	15 (9.1)	9 (11.0)	
Treatment				0.08
Monotherapy	134 (54.5)	96 (58.5)	38 (46.3)	
Combined immunological agents	41 (16.7)	25 (15.2)	16 (19.5)	
Immunotherapy + chemotherapy	32 (13.0)	17 (10.4)	15 (18.3)	
Immunotherapy + radiotherapy	26 (10.6)	20 (12.2)	6 (7.3)	
Other	13 (5.3)	6 (3.7)	7 (8.5)	
PD-L1 expression (TPS)				0.73
Low (0–49%)	169 (68.7)	111 (67.7)	58 (70.7)	
High (50–100%)	77 (31.3)	53 (32.3)	24 (29.3)	
Clinical stage				0.82
Limited stage	121 (49.1)	82 (50.0)	39 (47.6)	
Extensive stage	125 (51.9)	82 (50.0)	43 (52.4)	
Response status				0.31
Non-response	100 (40.7)	63 (38.4)	37 (45.1)	
Response	146 (59.3)	101 (61.6)	45 (54.9)	

LUAD, lung adenocarcinoma; LUSC, lung squamous cell carcinoma; TPS, tumor proportion score

### Feature selection

A total of 372 radiomic features were extracted from the imaging data based on each single habitat and the entire tumor. And 512 deep learning features were derived from the entire tumor. To reduce feature dimension and simplify model, we adopted a series of screening approach. After the screening using the ICC, Pearson correlation coefficients, two-sample t test, and the least absolute shrinkage selection operator regression method as depicted in Fig. 2c, optimal radiomic features were obtained from habitat 1, habitat 2, habitat3, habitat4, habitat 5, and the entire tumor. And deep learning features were selected based on the whole tumor.

### Performance evaluation of models

**Fig. 3:**
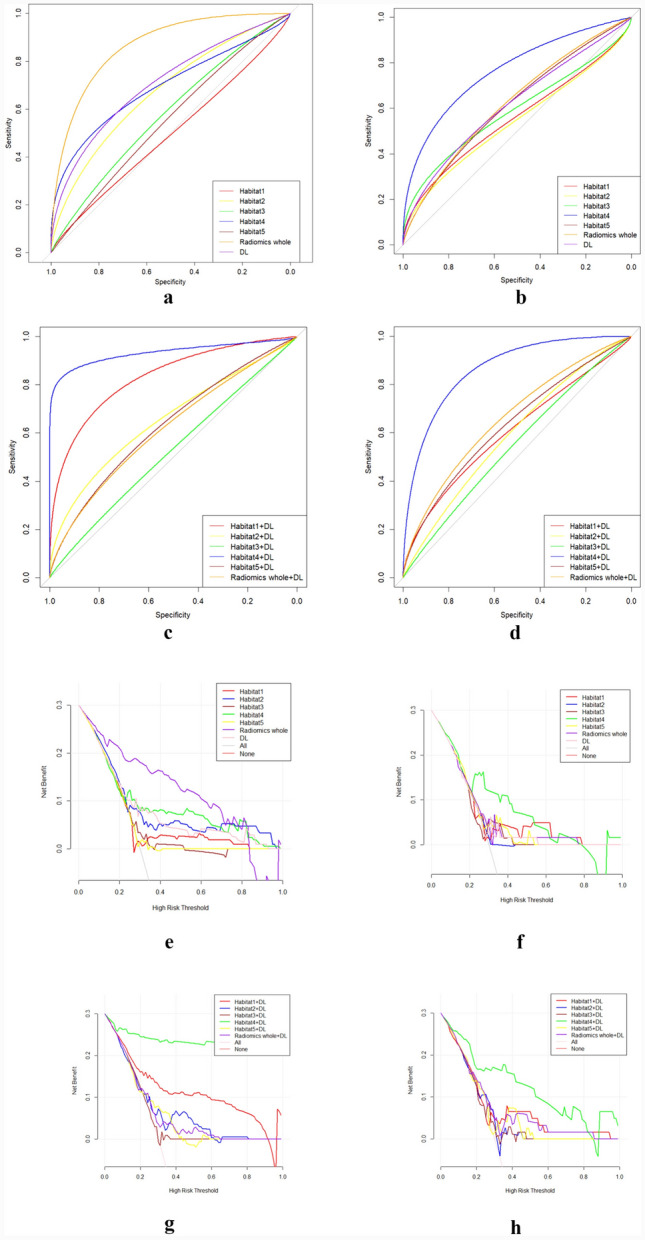
ROC curves of the SVM machine learning models in the training (**a**) and external test cohorts (**b**) based on single source features. The ROC curves of the SVM machine learning models in the training (**c**) and external test cohorts (**d**) based on combined features. The DCA curves of the SVM machine learning models in the training (**e**) and external test cohorts (**f**) based on single source features. The DCA curves of the SVM machine learning models in the training (**g**) and external test cohorts (**h**) based on combined features

The SVM machine learning algorithm was developed mainly based on two groups: One consisted of each single habitat, the entire tumor radiomic features, and the whole tumor deep learning features. And the other one is the combination between deep learning features and each radiomic features from different sub-regions, and combination of deep learning features and all habitat radiomics features. The prediction efficiency of each model is summarized in Table 2. Table 2 described the prediction performance of models based on different sub-regions, the entire tumor radiomic and deep learning features. Then we fused the deep learning features with radiomic features to construct novel machine learning models. Among of different combination, the deep learning features plus habitat 4 performed best as depicted as Fig. 3d, with AUC 0.865 (95% CI 0.772–0.931), accuracy 0.82, precision score 0.89, recall score 0.68, F1-score 0.77 in the external test cohort. Figure 3a–h demonstrated ROC, decision curve analysis of the different combinations in the training and test set, respectively.

**Table 2:** PFS6 prediction performance of SVM model based on single source features

	Task	Accuracy score	Precision score	Recall score	F1-score	AUC (95%CI)	MCC	PLR	NLR
Habitat1	Training	0.66	0.54	0.71	0.62	0.704 (0.628–0.772)	0.33	1.90	0.46
	Test	0.62	0.75	0.24	0.37	0.556 (0.443–0.666)	0.25	3.65	0.81
Habitat2	Training	0.61	0.49	0.75	0.59	0.667 (0.590–0.739)	0.27	1.57	0.48
	Test	0.65	0.79	0.30	0.43	0.541 (0.427–0.651)	0.31	4.46	0.75
Habitat3	Training	0.51	0.43	0.85	0.57	0.575 (0.495–0.651)	0.19	1.21	0.55
	Test	0.67	0.66	0.57	0.61	0.592 (0.478–0.700)	0.33	2.32	0.57
Habitat4	Training	0.70	0.61	0.63	0.62	0.691 (0.615–0.761)	0.37	2.47	0.49
	Test	0.77	0.78	0.68	0.72	0.781 (0.676–0.865)	0.53	4.34	0.38
Habitat5	Training	0.60	0.48	0.35	0.40	0.548 (0.469–0.626)	0.12	1.47	0.85
	Test	0.65	0.67	0.59	0.63	0.613 (0.499–0.719)	0.43	2.43	0.69
Whole	Training	0.82	0.77	0.76	0.77	0.874 (0.813–0.920)	0.63	5.50	0.28
	Test	0.65	0.64	0.49	0.55	0.613 (0.499–0.719)	0.28	2.19	0.66
DL	Training	0.66	0.54	0.71	0.62	0.704 (0.628–0.772)	0.33	1.90	0.46
	Test	0.67	0.64	0.62	0.63	0.631 (0.517–0.735)	0.33	2.15	0.53

PFS, progression-free survival; AUC, area under the receiver characteristic curve; MCC, Matthews Correlation Coefficient; PLR, positive likelihood ratio; NLR, negative likelihood ratio

**Fig. 4:**
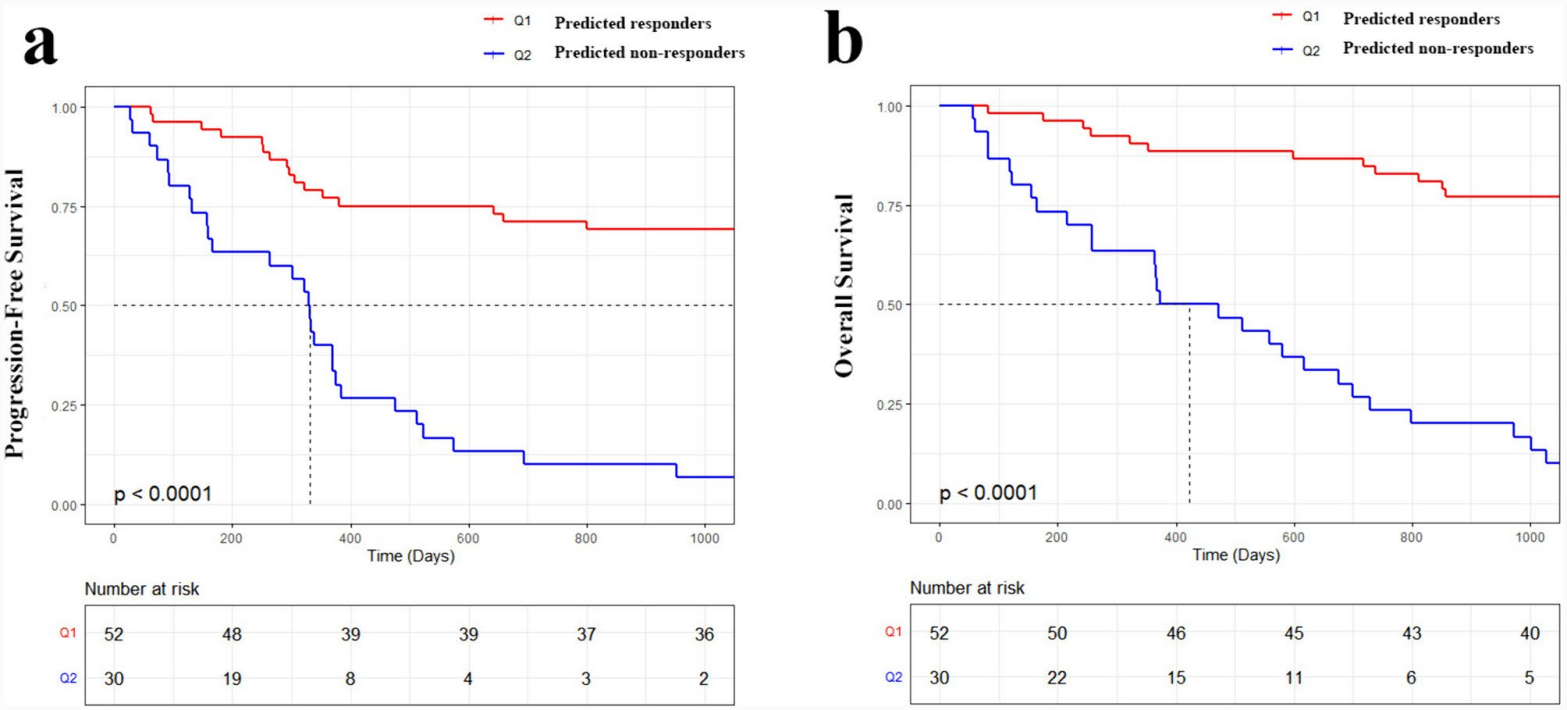
Kaplan–Meier survival curves on the independent test cohort for ensemble SVM models. **a** represents the PFS Kaplan–Meier curves; while, **b** represents the OS Kaplan–Meier curves

Then, we investigated the performance of the combination of habitat 4 radiomic features and deep learning features in predicting PFS and OS. As depicted in Fig. 4a–b, included individuals were grouped into high-risk and low-risk group based on the optimum cutoff of value generated by ROC curve in the both of training and test set. Figure 4 shows the Kaplan–Meier survival curves for PFS and OS on the independent test set for the combination SVM models. The combination SVM could significantly stratify PFS and OS for the clinical endpoint (*p* value < 0.05) (Table 3).

**Table 3:** PFS6 prediction performance of SVM model based on combined features

	Task	Accuracy score	Precision score	Recall score	F1-score	AUC (95%CI)	MCC	PLR	NLR
Habitat1 + DL	Training	0.72	0.59	0.90	0.71	0.827 (0.760–0.881)	0.33	1.90	0.46
	Test	0.67	0.86	0.32	0.47	0.607 (0.493–0.713)	0.25	3.65	0.81
Habitat2 + DL	Training	0.71	0.81	0.33	0.47	0.650 (0.572–0.723)	0.27	1.57	0.48
	Test	0.60	0.46	0.86	0.60	0.594 (0.480–0.701)	0.31	4.46	0.75
Habitat3 + DL	Training	0.62	0.50	0.22	0.31	0.521 (0.441–0.599)	0.19	1.21	0.55
	Test	0.56	0.43	0.78	0.56	0.554 (0.440–0.664)	0.33	2.32	0.57
Habitat4 + DL	Training	0.91	0.86	0.92	0.89	0.943 (0.895–0.973)	0.37	2.47	0.49
	Test	0.82	0.89	0.68	0.77	0.865 (0.772–0.931)	0.53	4.34	0.38
Habitat5 + DL	Training	0.67	0.57	0.57	0.57	0.652 (0.574–0.725)	0.12	1.47	0.85
	Test	0.67	0.78	0.38	0.51	0.627 (0.514–0.732)	0.43	2.43	0.69
All habitat + DL	Training	0.79	0.67	0.90	0.77	0.872 (0.811–0.919)	0.62	3.38	0.13
	Test	0.65	0.83	0.27	0.41	0.558 (0.445–0.668)	0.32	6.08	0.76
Whole + DL	Training	0.55	0.45	0.84	0.59	0.619 (0.540–0.694)	0.63	5.50	0.28
	Test	0.60	0.53	0.92	0.67	0.653 (0.539–0.754)	0.28	2.19	0.66

PFS, progression-free survival; SVM, support vector machine; AUC, area under the receiver characteristic curve; MCC, Matthews Correlation Coefficient; PLR, positive likelihood ratio; NLR, negative likelihood ratio

### Model explainability

**Fig. 5:**
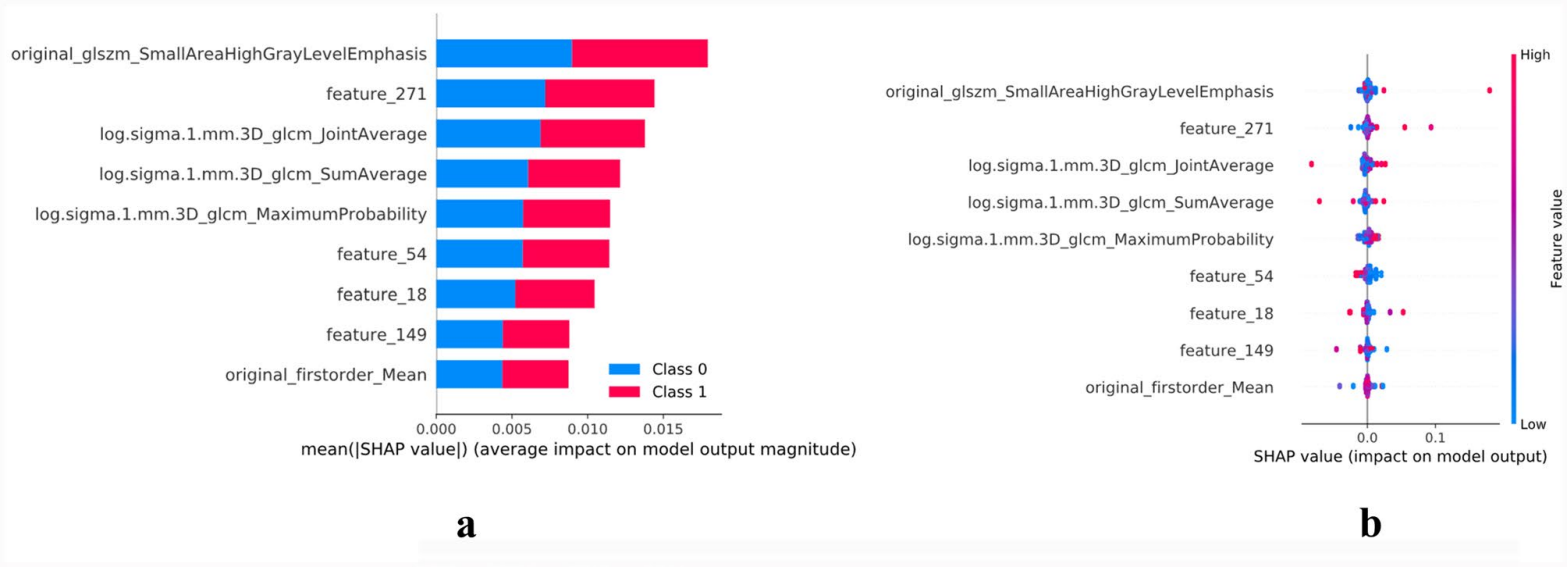
**a** Variable importance of the SVM model in the training set, showing that the original_glszm_SmallAreaHighGrayLevelEmphasis was the most important feature. **b** SHAP of the SVM model. The closer the values of the features were to 1, the more likely patients were to progress to be non-responders to the immunotherapy

The SHAP algorithm was utilized to illustrate the contribution of each feature to the model's final prediction. This algorithm was specifically applied to the model of the SVM, where a positive SHAP value indicated an elevated risk of disease progression for each prediction. As depicted in Fig. 5, the original_glszm_SmallAreaHighGrayLevelEmphasis and Feature 271 from deep learning extracted variable emerged as the most influential clinical variables.

## Discussion

Despite the immune checkpoint blockade has revolutionized treatment of NSCLC, a large number of patients remain non-responsive to the therapy [32]. Under these circumstances, there is an urgent need for a reliable signature to identify patients who may benefit from this advanced therapeutic approach. In this multi-institutional study, deep learning imaging features and radiomics based on habitat was employed to predict the clinical durable benefit at 6 months following the initiation of immune checkpoint inhibitor therapy in patients with NSCLC. The ensemble, which combines the deep learning features with the habitat radiomics, obtain the area under the curve (AUC) values of 0.943 (95% CI 0.895–0.973) training cohort and 0.865 (95% CI 0.772–0.931) for the external validation cohort.

CT is routinely applied in clinical settings to evaluate the immune therapy response to immune therapy in NSCLC patients [33]. Lesion diameters in the CT images could directly reflect tumor burden. There is growing evidence supporting the use of tumor diameters in CT scans to predict the responsivity to neoadjuvant chemotherapy and immune therapy [34, 35]. However, the immune therapy effect may precede any observable change of the tumor size [36]. Therefore, the reliance on mere superficial characteristics from CT image is insufficient for precise prediction of the therapy response. It necessitates the extraction of high-dimensional and high-throughput imaging features to accurately quantify the risk of prognosis in patients with NSCLC. This study underlined the need for more sophisticated imaging analytics in the evaluation of complex therapeutic outcomes.

When compared with conventional entire tumor radiomics, habitat radiomics have been considered as important for the prognosis [37, 38]. Moreover, the development of deep learning-based radiomics marks a significant advancement in the field, with its capacity to unearth deep-level imaging features not being recognized to the human eye [39], thereby offering novel perspectives on the prediction of the efficacy of immunotherapy. Deep learning's integration into conventional radiomics analysis of tumors is becoming increasingly important in the field of disease diagnosis, therapeutic strategy formulation, and prognostication [40–42].

To the best of our knowledge, no previous studies have revealed that if the integration of deep learning features and habitat radiomics features can stratify the patients at high risk of non-responsive to immune checkpoint inhibitor. Similarly, Vanguri et al. [21] previously reported that a multimodal model integrating baseline medical imaging, histopathological, and genomic features surpassed the performance of unimodal models achieving an AUC of 0.80 in predicting the response to immunotherapy.

Our study is subject to some limitations. First of all, the nature of retrospective and multicenter design of our study introduces heterogeneity within the cohort, particularly concerning treatment approaches and imaging protocols. Secondly, the interpretation of the deep features is often not straightforward since they are optimized to minimize the prediction error and are not designed to match human intuition or knowledge.

## Conclusion

In conclusion, an ensemble of deep learning features and habitat radiomics has been employed to predict the durable benefit of immunotherapy at 6 months after treatment. This suggests its potential as a viable non-invasive biomarker for drug responsivity prediction. The model's robust performance has been substantiated by a stable AUC in an external test cohort.

## Data Availability

The datasets used and/or analyzed during the current study are available from the corresponding author on reasonable request.
